# Research on Speed Estimation Method for Distributed Electric-Drive Loaders Based on Finite-State Machine

**DOI:** 10.3390/s26103168

**Published:** 2026-05-17

**Authors:** Xinyu Qi, Yalei Liu, Xiaohan Yuan, Yongqing Yuan, Mingliang Yang

**Affiliations:** 1 School of Mechanical Engineering, Southwest Jiaotong University, Chengdu 610031, China; qixinyu2024@my.swjtu.edu.cn (X.Q.); liuyl2017@my.swjtu.edu.cn (Y.L.); 2 Shanghai Cosmic Automotive Technology Co., Ltd., Shanghai 201800, China; yuanxiaohan@mychery.com (X.Y.); yuanyongqing@mychery.com (Y.Y.)

**Keywords:** distributed electric-drive loader, speed estimation, Finite State Machine, multi-sensor fusion

## Abstract

**Highlights:**

**What are the main findings?**

- FSM-Based Adaptive Fusion Strategy.
- Compensation for Articulated Steering Kinematics.

**What are the implications of the main findings?**

- Theoretical Advancement: Provides a novel framework for articulated vehicle state estimation under extreme slip, advancing coupled dynamics and sensor interference handling.
- Engineering Application Value: Delivers high-accuracy speed signals for traction control, enhancing loader performance and safety on challenging terrains.

**Abstract:**

Speed information is crucial for controlling distributed electric-drive loaders, especially for driving and operation. Due to complex working conditions, the wheels of the loader often experience different conditions, leading to inaccurate speed estimation. To solve this, this paper proposes a multi-sensor fusion speed estimation method based on a Finite State Machine (FSM). The method uses the FSM to identify the wheel states and adaptively switches between the weighted average method and integration method to estimate the vehicle’s speed accurately. When all wheels are slipping, the acceleration integration method is used, starting from the latest trustworthy speed estimate. When the wheels are not slipping, the speed is estimated using the weighted average of the trustworthy wheels. Additionally, the method addresses the relative motion between the front and rear vehicle bodies caused by articulated steering by using an articulated steering projection method to ensure accurate wheel state estimation from IMU signals. Simulation and hardware-in-the-loop experiments show that the proposed method can accurately estimate vehicle speed under various road conditions. Specifically, under low-adhesion road conditions with all four wheels in a slipping state, it improves speed estimation accuracy by over 75% compared to traditional methods such as simple averaging, selective averaging, and pure integration.

## 1. Introduction

In recent years, driven by the “dual carbon” strategic goals, energy-saving and emission-reduction policies have created new opportunities for the electrification transformation and development of the construction machinery industry, while also presenting new challenges [1,2,3]. As an important type of construction machinery, loaders have a large market share and are widely used in various scenarios such as ports, construction sites, and stone quarries. Compared to traditional diesel-powered loaders, distributed electric-drive loaders [4,5,6] can achieve independent drive for each axle and wheel, offering flexible chassis configuration, a short drive chain, and high transmission efficiency. Through proper control, they can effectively enhance overall machine performance under complex working conditions [7,8].

The operating conditions of loaders are generally complex and harsh, with significant variations in the road surface adhesion coefficient. During work and travel, the front and rear loads change drastically, and during digging, the wheels may even become airborne [9,10]. Due to the structural characteristics of distributed electric-drive loaders, which feature four-wheel drive and no passive wheels, combined with their operation in complex conditions and harsh environments, traditional speed estimation methods face significant challenges [11]. Vehicle speed information is a core state parameter in vehicle dynamics control. Therefore, accurate speed estimation is of paramount importance for distributed electric-drive loaders.

Many methods have been proposed to accurately estimate the vehicle speed [12]. Currently, research in the field of longitudinal speed estimation has predominantly focused on passenger and commercial vehicles. However, for specialized construction machinery such as loaders, a mature and systematic vehicle speed estimation methodology has not yet been established. Typical methods for longitudinal speed estimation include those based on wheel speed information, where the wheel speed is calculated from the feedback rotational speed of the drive motor, and the average of the four wheel speeds is used as the estimated vehicle speed. While this method is simple and cost-effective, its accuracy significantly decreases when wheel slip occurs.Building on this, the threshold-based selective averaging method was proposed, which improves estimation accuracy by weighting the wheel speeds of non-slipping wheels. However, in extreme conditions where all four wheels are slipping, this method suffers from a significant deviation from the true value due to the loss of valid references. Another approach involves using the Inertial Measurement Unit (IMU) to integrate longitudinal acceleration in order to obtain vehicle speed [13,14]. Although this method is suitable for four-wheel-drive vehicles, it suffers from the issue of integration error accumulation, which affects long-term estimation accuracy. Other studies have designed vehicle speed estimators using wheel speed sensor signals and Inertial Measurement Unit (IMU) data, while also utilizing Global Positioning System (GPS) data for calibration [15]. However, conventional GPS has limited accuracy, and sensor signals are susceptible to environmental interference [16]. Especially for loaders operating in signal-limited environments such as mining areas and material yards, GPS signals are prone to blockage or multipath effects, leading to insufficient estimation stability. Currently, some vehicle speed estimation methods adopt observer-based strategies or machine learning approaches [17,18], achieving promising results on regular on-road vehicles. These approaches can partially handle nonlinear system noise and model uncertainties, thereby improving estimation accuracy and robustness. However, they often require substantial computational resources and rely on extensive calibration data for model training, which limits their direct application in embedded real-time control systems.

Given the limitations of existing research, this paper adopts the concept of Finite State Machine (FSM) and designs an adaptive switching mechanism for the speed estimation strategy within the trustworthy wheel speed identification module. A longitudinal speed estimation method for distributed electric-drive loaders based on FSM multi-sensor fusion is proposed. The method mainly consists of four core components: wheel speed/IMU signal processing, trustworthy wheel speed identification, and longitudinal vehicle speed estimation. By comprehensively considering the validity of wheel motion states and their rotational speed information, and integrating longitudinal acceleration data, it achieves accurate estimation of the longitudinal speed of the loader.

## 2. Overall Longitudinal Speed Estimation Scheme

**Figure 1:**
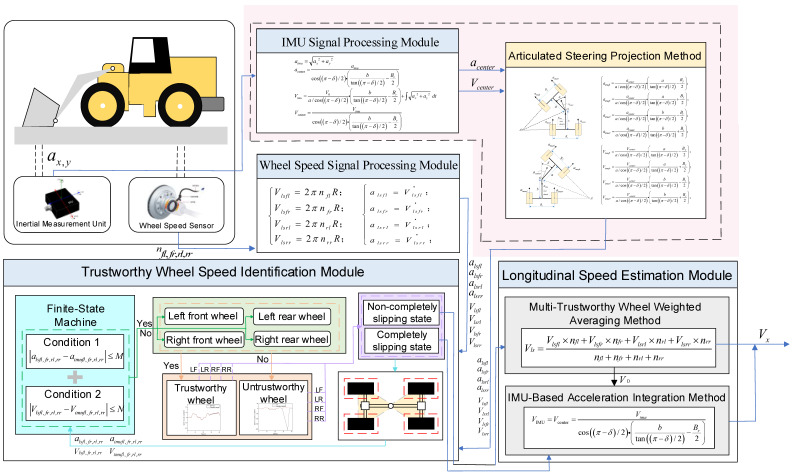
The Overall Process of Vehicle Speed Estimation for Distributed Electric-Drive Loaders.

The overall process of the longitudinal speed estimation method proposed in this paper is shown in Figure 1. It mainly includes four core modules: wheel speed signal processing, IMU signal processing, trustworthy wheel speed identification, and longitudinal speed estimation. In the figure, $n_{fl}$, $n_{fr}$, $n_{rl}$, $n_{rr}$ represent the rotational speeds of the left front wheel, right front wheel, left rear wheel, and right rear wheel obtained from the wheel speed sensor signals; $a_{x}$, $a_{y}$ represent the longitudinal and lateral accelerations at the IMU installation location, i.e., the rear chassis, both of which can be measured by the loader’s inertial sensors; δ represents the articulation angle of the loader at the articulation point; $V_{lsfl}$, $V_{lsfr}$, $V_{lsrl}$, $V_{lsrr}$ represent the linear velocities of the left front wheel, right front wheel, left rear wheel, and right rear wheel, respectively, calculated from the wheel speed sensor signals; $a_{lsfl}$, $a_{lsfr}$, $a_{lsrl}$, $a_{lsrr}$ represent the accelerations of the left front wheel, right front wheel, left rear wheel, and right rear wheel, respectively, calculated from the wheel speed sensor signals; $V_{0}$ represents the initial velocity of the integration method, selected as the last accurate longitudinal speed estimation value when not all four wheels are slipping; $a_{imufl}$, $a_{imufr}$, $a_{imurl}$, $a_{imurr}$ represent the accelerations of the left front wheel, right front wheel, left rear wheel, and right rear wheel, respectively, calculated from the IMU signals; $V_{imufl}$, $V_{imufr}$, $V_{imurl}$, $V_{imurr}$ represent the linear velocities of the left front wheel, right front wheel, left rear wheel, and right rear wheel, respectively, calculated from the IMU signals.

In the wheel speed signal processing module, the rotational speed signals measured by the four wheel speed sensors are processed to calculate the speed and acceleration of each wheel. The IMU signal processing module processes the signals from the Inertial Measurement Unit (IMU), and in this step, the articulated steering projection method proposed in this paper is introduced to accurately solve the longitudinal speed of the vehicle’s center of mass and the reference acceleration of each wheel. In the trustworthy wheel speed identification module, the system combines the processing results from the previous two modules and, based on the obtained vehicle dynamic state information, determines the slip state of the four wheels. Finally, the longitudinal speed estimation module, based on the wheel slip states output by the trustworthy wheel speed identification module, adaptively selects the optimal estimation algorithm and outputs a high-precision longitudinal speed estimate for the loader.

## 3. Longitudinal Speed Estimation Method

### 3.1. Wheel Speed Signal Processing Module

Wheel speed sensors are typically installed on the wheels and can measure the rotational speed of the wheels. The linear velocity of each wheel can be calculated using Equation (1), and by differentiating this, the acceleration can be obtained as shown in Equation (2):

(1) $$\left\{\begin{array}{c} V_{lsfl}=2\pi n_{fl}R\\ V_{lsfr}=2\pi n_{fr}R\\ V_{lsrl}=2\pi n_{rl}R\\ V_{lsrr}=2\pi n_{rr}R \end{array}\right.$$

(2) $$\left\{\begin{array}{c} a_{lsfl}=\overset{.}{V_{lsfl}}\\ a_{lsfr}=\overset{.}{V_{lsfr}}\\ a_{lsrl}=\overset{.}{V_{lsrl}}\\ a_{lsrr}=\overset{.}{V_{lsrr}} \end{array}\right.$$

In the equation, R represents the rolling radius of the loader’s wheel.

### 3.2. IMU Signal Processing Module

**Figure 2:**
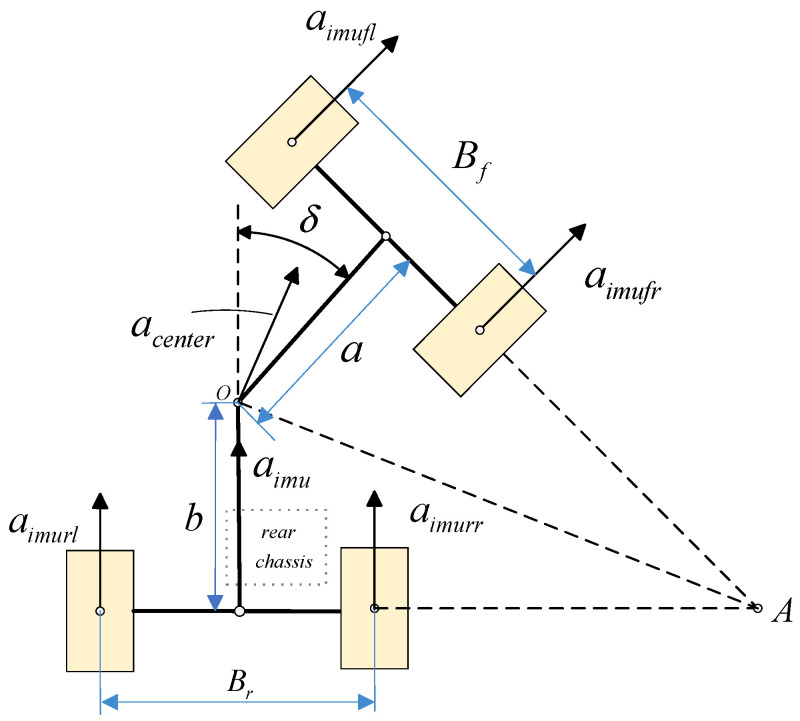
Articulated Steering Projection Method (Right Turn).

**Figure 3:**
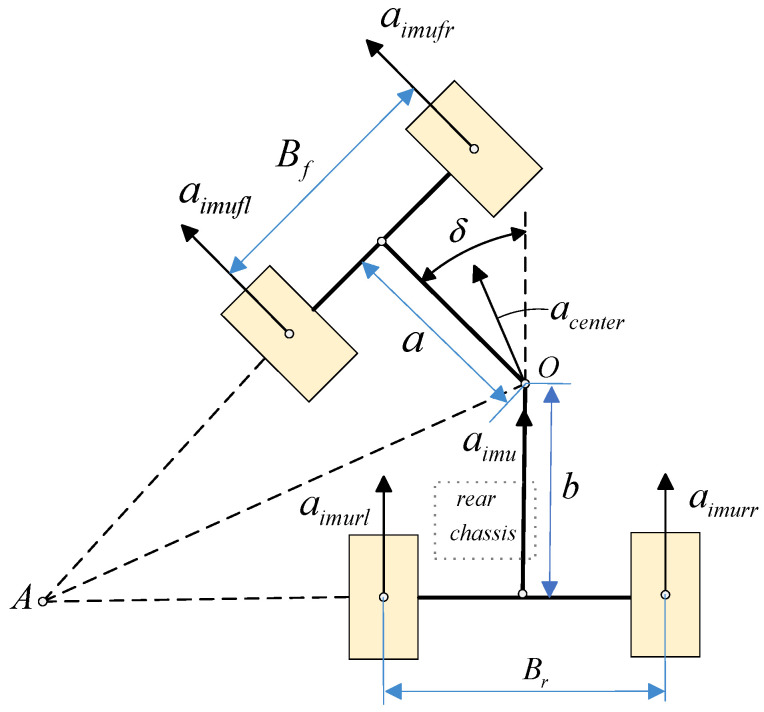
Articulated Steering Projection Method (Left Turn).

The loader adopts an articulated steering structure, where the relative deflection between the front and rear chassis is achieved through a central articulation pin. This structure causes the acceleration measured by the IMU, mounted on the rear chassis, to only represent the motion of the rear chassis itself, rather than the true motion direction of the vehicle’s center of mass. Direct integration of this local acceleration to estimate vehicle speed would result in significant estimation errors. To address this, this paper proposes the articulated steering projection method, which is based on introducing real-time articulation angle information to establish the kinematic projection relationship from the IMU installation location to the vehicle’s center of mass and the four wheels. This method accurately converts the local observations of the rear chassis into the global motion state of the entire vehicle. This approach provides a unified and reliable theoretical basis for the subsequent trustworthy wheel speed identification module, based on multi-source information fusion. A schematic of the loader’s articulated steering structure is shown in Figure 2 and Figure 3.

Taking the loader’s left turn as an example, the acceleration and velocity at the installation location obtained from the IMU signal [19] can be expressed by Equations (3) and (4) as shown

(3) $$a_{imu}=\sqrt{{a_{x}}^{2}+{a_{y}}^{2}}$$

(4) $$V_{imu}=\frac{V_{0}\times \cos\left(\frac{\pi -\delta }{2}\right)}{a}\times \left(\frac{b}{\tan\left(\frac{\pi -\delta }{2}\right)}-\frac{B_{r}}{2}\right)+\int \sqrt{{a_{x}}^{2}+{a_{y}}^{2}}\;dt$$

In the equation, $a_{imu}$ represents the acceleration calculated by the integration method at the IMU sensor installation location, and $V_{imu}$ represents the velocity calculated by the integration method at the IMU sensor installation location, i.e., the rear chassis. Through geometric relationship analysis, the acceleration and velocity of the four wheels can be obtained:

(5) $$a_{\mathrm{center}}=\frac{a_{imu}}{\cos\left(\frac{\pi -\delta }{2}\right)}\;\times \;\;\frac{1}{\left(\frac{b}{\tan\left(\frac{\pi -\delta }{2}\right)}-\frac{B_{r}}{2}\right)}$$

(6) $$\left\{\begin{array}{c} a_{imufl}=\frac{a_{center}\cos\left(\frac{\pi -\delta }{2}\right)}{a}\times \left(\frac{a}{\tan\left(\frac{\pi -\delta }{2}\right)}-\frac{B_{f}}{2}\right)\\ a_{imufr}=\frac{a_{center}\cos\left(\frac{\pi -\delta }{2}\right)}{a}\times \left(\frac{a}{\tan\left(\frac{\pi -\delta }{2}\right)}+\frac{B_{f}}{2}\right)\\ a_{imurl}=\frac{a_{center}\cos\left(\frac{\pi -\delta }{2}\right)}{a}\times \left(\frac{b}{\tan\left(\frac{\pi -\delta }{2}\right)}-\frac{B_{r}}{2}\right)\\ a_{imurr}=\frac{a_{center}\cos\left(\frac{\pi -\delta }{2}\right)}{a}\times \left(\frac{b}{\tan\left(\frac{\pi -\delta }{2}\right)}+\frac{B_{r}}{2}\right) \end{array}\right.$$

(7) $$V_{\mathrm{center}}=\frac{V_{imu}}{\cos\left(\frac{\pi -\delta }{2}\right)\times \left(\frac{b}{\tan\left(\frac{\pi -\delta }{2}\right)}-\frac{B_{r}}{2}\right)}$$

(8) $$\left\{\begin{array}{c} V_{imufl}=\frac{V_{center}\;\cos\left(\frac{\pi -\delta }{2}\right)}{a}\times \left(\frac{a}{\tan\left(\frac{\pi -\delta }{2}\right)}-\frac{B_{f}}{2}\right)\\ V_{imufr}=\frac{V_{center}\;\cos\left(\frac{\pi -\delta }{2}\right)}{a}\times \left(\frac{a}{\tan\left(\frac{\pi -\delta }{2}\right)}+\frac{B_{f}}{2}\right)\\ V_{imurl}=\frac{V_{center}\;\cos\left(\frac{\pi -\delta }{2}\right)}{a}\times \left(\frac{b}{\tan\left(\frac{\pi -\delta }{2}\right)}-\frac{B_{r}}{2}\right)\\ V_{imurr}=\frac{V_{center}\;\cos\left(\frac{\pi -\delta }{2}\right)}{a}\times \left(\frac{b}{\tan\left(\frac{\pi -\delta }{2}\right)}+\frac{B_{r}}{2}\right) \end{array}\right.$$

In the equation, a, b represent the distances from the center of mass to the front axle and rear axle, respectively; $B_{f}$, $B_{r}$ represent the front axle and rear axle track widths, respectively; $a_{\mathrm{center}}$ is the acceleration at the center of mass; and $V_{\mathrm{center}}$ is the velocity at the center of mass.

The above analysis assumes ideal two-dimensional planar motion, where longitudinal speed is derived directly from IMU signals. However, pitch and roll angles cause gravity components along each axis, introducing projection errors. Thus, under large pitch/roll conditions, the two-dimensional projection leads to non-negligible systematic errors. The proposed method targets longitudinal speed estimation on horizontal roads. For scenarios with strong attitude variations, an online gravity compensation method using motion constraints is recommended.

### 3.3. Trustworthy Wheel Speed Identification Module

**Figure 4:**
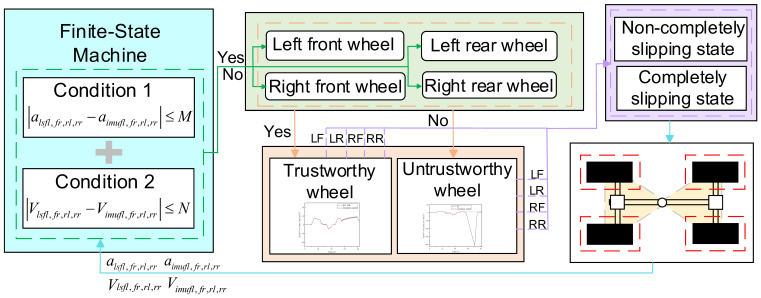
Finite State Machine for Trustworthy Wheel Speed Identification.

This paper introduces the concept of FSM [20] in the trustworthy wheel speed identification module, utilizing its “state transition” and “event-triggered” mechanisms [21].An adaptive speed estimation strategy switching mechanism is designed, as shown in Figure 4. This design enhances the robustness of recognizing the dynamic changes in the tire slip state by incorporating the concept of time-domain analysis in the state judgment.

To accurately determine the tire state of the loader, this paper uses two conditions to assess the reliability of the wheel speed. The first condition is that the absolute value of the difference between the wheel acceleration obtained from the wheel speed sensor signals and the wheel acceleration obtained from the IMU signals is less than a set threshold M; the second condition is that the absolute value of the difference between the wheel linear velocity obtained from the wheel speed sensor signals and the longitudinal velocity obtained from the IMU signals is less than a set threshold N. The thresholds M and N are determined through calibration based on experimental data and the loader model. When a wheel satisfies both conditions simultaneously, it is considered that the wheel is not slipping, and its wheel speed signal is deemed reliable and can be regarded as a trustworthy wheel. This state is represented by the following formulas, as shown in Equations (9)–(12):

(9) $$\left|a_{lsfl}-a_{imufl}\right|\leq M\cap \left|V_{lsfl}-V_{imufl}\right|\leq N$$

(10) $$\left|a_{lsfr}-a_{imufr}\right|\leq M\cap \left|V_{lsfr}-V_{imufr}\right|\leq N$$

(11) $$\left|a_{lsrl}-a_{imurl}\right|\leq M\cap \left|V_{lsrl}-V_{imurl}\right|\leq N$$

(12) $$\left|a_{lsrr}-a_{imurr}\right|\leq M\cap \left|V_{lsrr}-V_{imurr}\right|\leq N$$

Conversely, it is considered a non-trustworthy wheel, as shown in Equations (13)–(16):

(13) $$\left|a_{lsfl}-a_{imufl}\right|>M\cup \left|V_{lsfl}-V_{imufl}\right|>N$$

(14) $$\left|a_{lsfr}-a_{imufr}\right|>M\cup \left|V_{lsfr}-V_{imufr}\right|>N$$

(15) $$\left|a_{lsrl}-a_{imurl}\right|>M\cup \left|V_{lsrl}-V_{imurl}\right|>N$$

(16) $$\left|a_{lsrr}-a_{imurr}\right|>M\cup \left|V_{lsrr}-V_{imurr}\right|>N$$

Considering that the vehicle may be in a critical state between slipping and non-slipping, a fixed-length time window is set to statistically count the number of trustworthy wheels at each moment within the window, determining the overall slipping state of the vehicle in that interval. The window length is set to 0.5 s, which is determined as the optimum through extensive tuning and repeated testing, because the transition of loader wheels from adhesion to slipping typically occurs within 0.2–0.5 s. A shorter window would be susceptible to noise and cause frequent state switching, while a longer window would delay the response. When a sharp change in the wheel speed or IMU signal is detected (which may indicate the beginning of slipping), the window is temporarily shortened for a quick response; when the vehicle is in a steady state or on a noisy, bumpy road surface, the window is restored to ensure stability. If there are trustworthy wheels within the window, the vehicle is considered to be in a “non-completely slipping state”; if no trustworthy wheels exist at any moment within the window, the vehicle is considered to be in a “completely slipping state.” The corresponding state transition logic, defined in terms of current state, event, and next state, is summarized in Table 1.

### 3.4. Longitudinal Speed Estimation Module

Based on the trustworthy wheel speed identification module described in the previous section, the system can accurately determine two operational states of the loader: “non-completely slipping state” and “completely slipping state.” This provides a reliable basis for the subsequent speed estimation strategy and effectively addresses the interference caused by slipping under complex working conditions.

When the system detects the non-completely slipping state, it will automatically switch to a multi-trustworthy wheel weighted average speed estimation based on wheel speed sensor information. By performing a weighted fusion of the wheel speeds of non-slipping wheels, the estimated longitudinal speed of the entire vehicle is obtained. The principle is shown in Equation (17). This strategy fully utilizes valid wheel speed information and is suitable for driving conditions under normal adhesion levels.

(17) $$V_{ls}=\frac{V_{lsfl}\times n_{fl}+V_{lsfr}\times n_{fr}+V_{lsrl}\times n_{rl}+V_{lsrr}\times n_{rr}}{n_{fl}+n_{fr}+n_{rl}+n_{rr}}$$

In the completely slipping state, an acceleration integration-based estimation algorithm using the Inertial Measurement Unit (IMU) is applied. This algorithm uses the trustworthy speed estimate from the previous time step as the initial value for integration and obtains the current longitudinal speed by real-time integration of the acceleration signal. The principle is shown in Equation (7), which effectively avoids estimation interruptions caused by the failure of wheel speed sensor information. At the same time, to address the inherent limitation of error accumulation in the acceleration integration method, the proposed estimation scheme is only used when all four wheels are completely slipping within the time domain. The integration time is kept short, and the initial value for each integration is updated, thereby improving the accuracy of the speed estimate.

## 4. Simulation Testing and Validation

**Figure 5:**
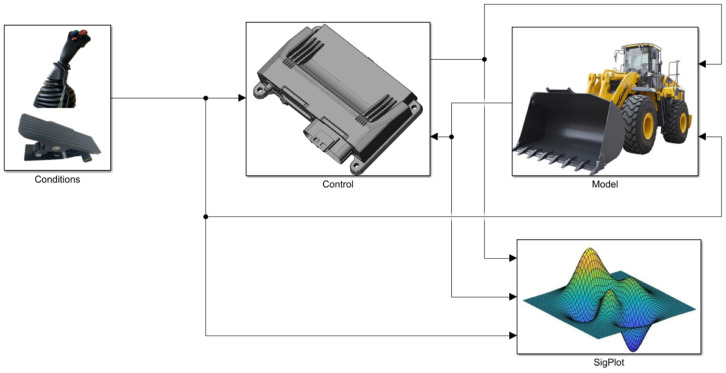
Full−Vehicle Dynamic Simulation Model.

To verify the effectiveness and adaptability of the longitudinal speed estimation method for distributed electric-drive loaders proposed in this paper, a full-vehicle dynamic simulation model was established in the MATLAB R2022a/Simscape environment, as shown in Figure 5. The tests were conducted using a typical loader V-type operation cycle, performed under two typical conditions: high-adhesion surfaces and low-adhesion surfaces, to comprehensively evaluate the performance of the algorithm under different slipping risks.

For comparative analysis, three typical vehicle speed estimation methods were selected as benchmarks: the simple average method based on the four-wheel wheel speed (TA), the selective averaging method based on threshold that weights the trustworthy wheel speeds after detecting the slipping state (WAT), and the estimation method based on IMU acceleration integration (IA). The TA method represents the most basic approach without fault tolerance, WAT reflects an improvement strategy based on wheel speed information, and the IA method, which uses a different physical sensing mechanism from wheel speed sensors, addresses the issue that the previous two methods cannot estimate speed when all four wheels are slipping. The FSM-based multi-sensor fusion method proposed in this paper (MSF-FSM) is an optimization of the aforementioned three methods. A comparison between these methods and the proposed method is conducted to verify the superiority of the proposed approach.

### 4.1. Loader V-Type Operating Cycle

**Figure 6:**
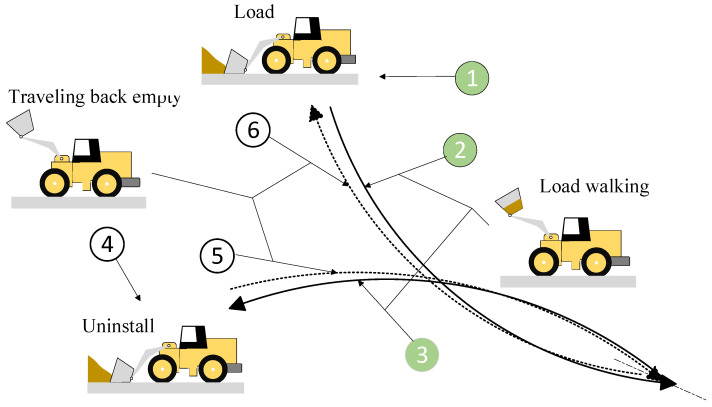
Schematic Diagram of the V−Type Operating Cycle.

The entire process of the typical loader V-type operating cycle can be divided into four stages: bucket loading, loading travel, unloading, and empty return travel, as shown in Figure 6. In the loading stage, the loader moves slowly at the material pile, performing operations such as bucket insertion, scooping, and lifting. In the loading travel stage, after loading is completed, the loader moves to the truck position by reversing and moving forward. This stage includes starting, towing, and steering, while the working device lifts and prepares the bucket for unloading. In the unloading stage, the loader remains stationary and inverts the bucket to unload the material into the truck, with no movement of the travel system. In the empty return travel stage, after unloading, the loader moves back to the material pile position by reversing and moving forward, while also leveling the bucket and lowering the boom, preparing for the next cycle.

### 4.2. Simulation Validation Under High-Adhesion Surface Conditions

**Figure 7:**
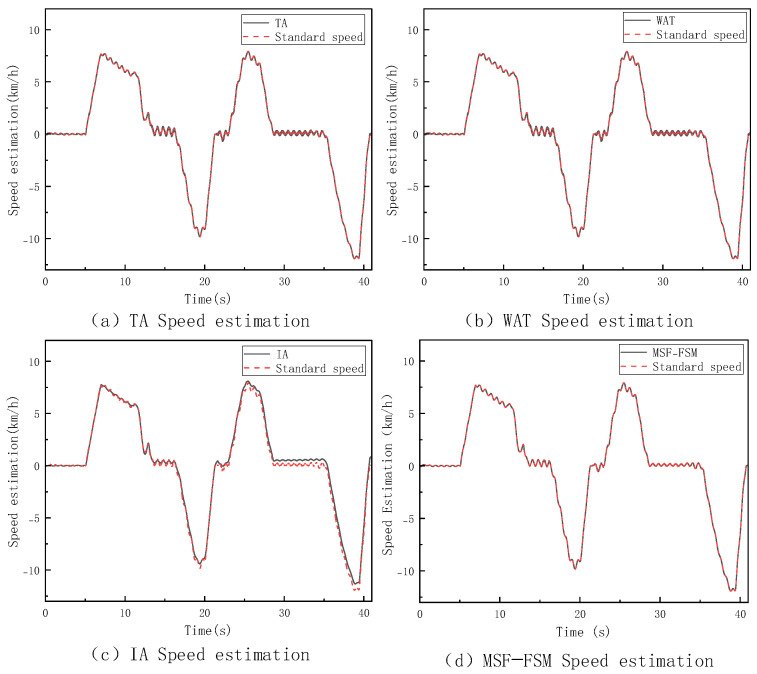
Vehicle Speed Estimation Method on High−Adhesion Surfaces.

**Figure 8:**
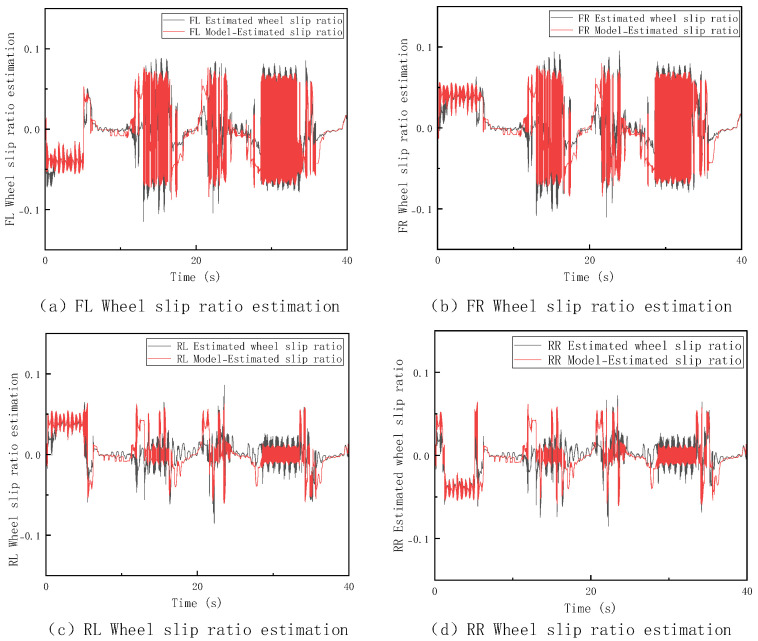
Slip Ratio Estimation on High−Adhesion Surfaces.

In this section, a V-type operation cycle driving test is conducted on the loader under high-adhesion surface conditions with a coefficient of adhesion of 0.9, and the corresponding results are shown in Figure 7. To further assess the performance of the speed estimation method and cross-validate with the aforementioned slipping criteria, Figure 8 further displays the dynamic changes in the slip ratios of each wheel under these conditions. The thresholds for trustworthy wheel determination are set to $M=0.1\;{m/s}^{2}$ and N=0.3 m/s through repeated calibration. To evaluate their sensitivity, we fixed N=0.3 and tested M=0.01,0.05,0.08,0.1,0.12,0.15,0.2. The estimation error remains low and stable when M is within 0.08–0.12, with the baseline M=0.1 at the center; the error increases significantly when M<0.08 or M>0.12. The same analysis applies to N. Thus, the selected thresholds are robust under the tested conditions. Future work may introduce adaptive thresholds to improve generalization.

As can be seen from the figure, under high-adhesion conditions, the estimation results of the three methods—TA, WAT, and MSF-FSM—are highly consistent with the true vehicle speed. This is mainly because, in this case, the tires have good adhesion to the ground, and the wheels experience little to no slip, with the wheel speed signal being very close to the true value. All three of these methods are based on averaging the wheel speed signals, so they maintain high accuracy. In contrast, the IA method, due to the error accumulation effect in the acceleration integration process, shows that its estimation results gradually deviate from the true value over time.

The introduction of slip ratio analysis provides direct experimental evidence for the effectiveness of the “Trustworthy Wheel Speed Identification Module.” As seen in the figure above, the system is indeed in a “non-completely slipping state,” consistent with the judgment logic of the MSF-FSM method. This method selects a multi-trustworthy wheel weighted average speed estimation based on wheel speed sensor information. This indicates that the method can choose the optimal estimation approach based on the actual contact between the wheels and the ground, ensuring the reliability and stability of the estimation results under various operating conditions.

### 4.3. Simulation Validation Under Low-Adhesion Surface Conditions

Under low-adhesion surface conditions, significant wheel slip between the tires and the ground can occur, severely affecting the accuracy of wheel speed signal readings. Therefore, the robustness of the speed estimation algorithm under slipping conditions becomes a key metric for evaluating its engineering applicability. To validate the performance of the proposed method under complex conditions, a typical V-type operating cycle with an adhesion coefficient of 0.35 was constructed. By comparing with the three traditional estimation methods—TA, WAT, and IA—the speed estimation accuracy and robustness of each algorithm under different levels of slip were systematically evaluated.

#### 4.3.1. Non-Four-Wheel Full Slip Condition

**Figure 9:**
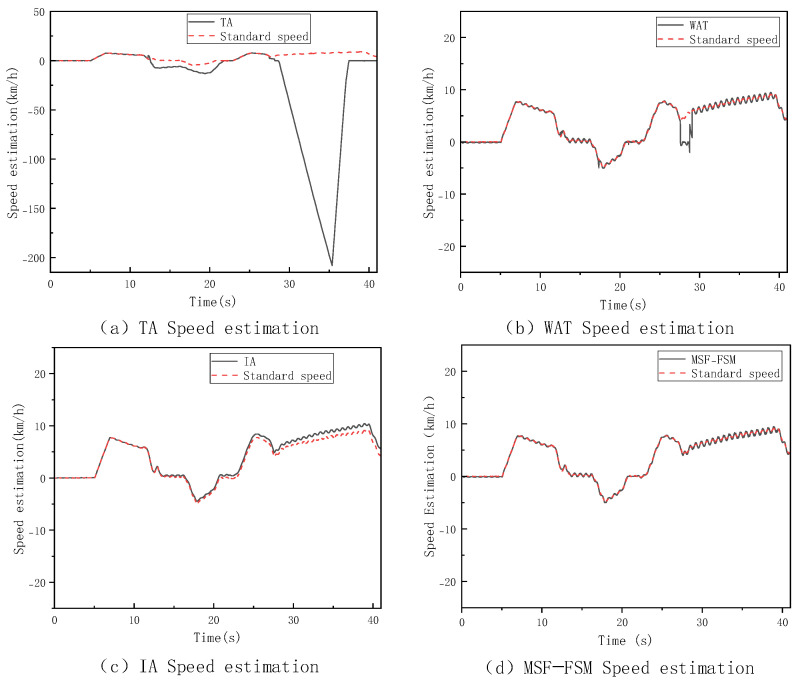
Vehicle Speed Estimation under Singl−Wheel Slip Condition on Low-Adhesion Surfaces.

**Figure 10:**
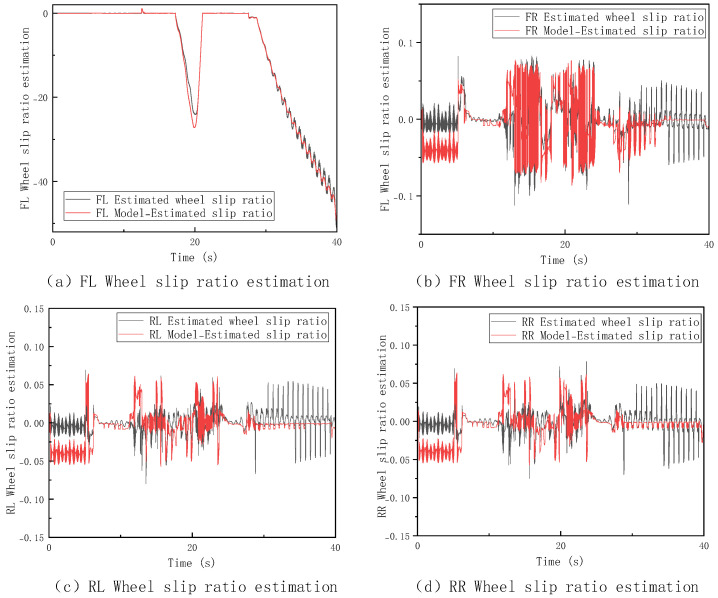
Slip Ratio Estimation under Singl−Wheel Slip Condition on Low-Adhesion Surfaces.

During the V-type operation cycle test on a low-adhesion surface with an adhesion coefficient of 0.35, this study set up various conditions, including single-wheel, dual-wheel, and three-wheel slipping. Since the estimation performance of different methods under these conditions exhibited similar trends and strong consistency, this paper selects the single-wheel slipping condition for analysis. The corresponding vehicle speed estimation results and slip ratio changes are shown in Figure 9 and Figure 10.

By combining the vehicle speed estimation and slip ratio estimation graphs, it is clear that under the single-wheel slip condition, the four methods show significant differences in performance. The TA method, due to its failure to effectively identify slipping wheel speeds, directly incorporates abnormal wheel speed information into the calculation, causing its estimation to deviate significantly from the true vehicle speed. The WAT method, while able to identify and exclude some abnormal wheel speeds, experiences delays and uncertainties in its judgment mechanism during state transitions or slip critical points, resulting in phase estimation errors. The IA method provides smoother outputs in the short term, but as the experiment progresses, the integration error gradually becomes more apparent. In contrast, the MSF-FSM method proposed in this paper dynamically fuses trustworthy wheel speeds and IMU information via the finite state machine, effectively eliminating unreliable wheel speeds during slip events, and maintaining optimal estimation accuracy throughout the process. To further quantify the comparison, Table 2 lists the specific values for maximum error, average error and RMSE for the four methods.

It can be observed that the TA method, due to incorporating invalid slipping wheel speed data, results in a significantly larger estimation error. Although the WAT and IA methods perform relatively well in terms of average error, their maximum errors are larger. The MSF-FSM method proposed in this paper outperforms the other comparison methods in both maximum error and average error, demonstrating superior estimation stability and robustness under varying operating conditions. The RMSE of the proposed MSF-FSM method is only 0.17, which is substantially better than that of TA (38.78), as TA directly averages all wheel speeds without excluding invalid data from the slipping wheel, leading to severe error fluctuations. Compared with WAT (0.97) and IA (0.70), MSF-FSM also achieves a lower RMSE, demonstrating more stable temporal estimation and smaller dispersion. Thus, even under single-wheel slip conditions, the proposed method maintains high smoothness and consistency in speed estimation.

#### 4.3.2. Four-Wheel Slip Condition

**Figure 11:**
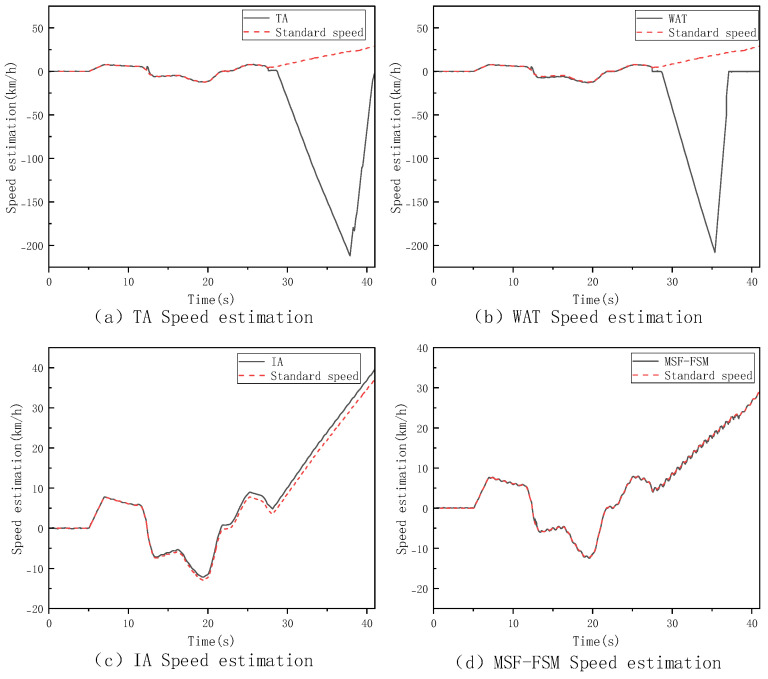
Vehicle Speed Estimation under Four−Wheel Slip Condition on Low-Adhesion Surfaces.

**Figure 12:**
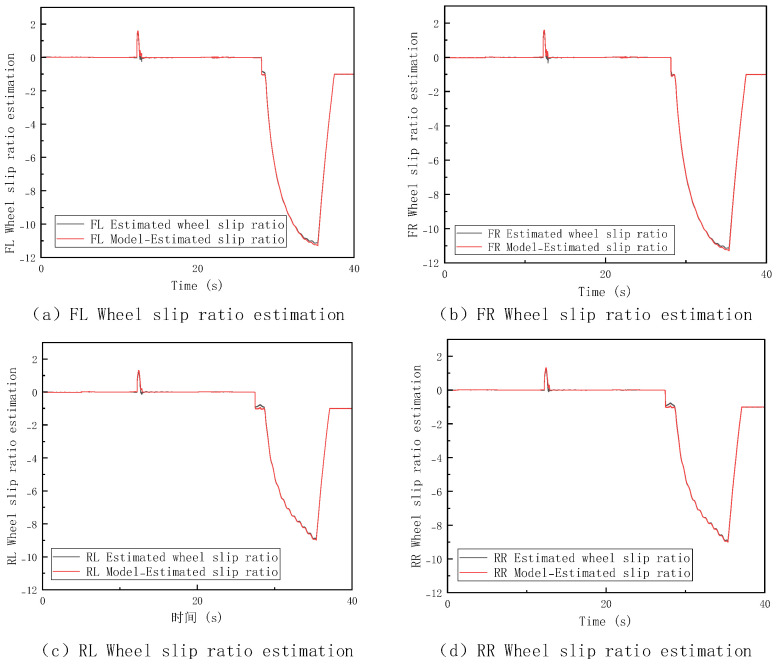
Slip Ratio Estimation under Four−Wheel Slip Condition on Low-Adhesion Surfaces.

The loader was tested under a V-type operation cycle on a low-adhesion surface with an adhesion coefficient of 0.35, further setting up a condition where all four wheels experience slip. The corresponding vehicle speed estimation results and slip ratio changes are shown in Figure 11 and Figure 12.

In the most extreme condition of four-wheel full slip, combining the analysis of Figures, when all four wheel slip ratios change sharply at the same time, all wheels are slipping, and the wheel speed signals completely lose their ability to represent the true vehicle speed. The TA and WAT methods, which rely on wheel speed signals at their core, cannot obtain valid wheel speed information under this condition, resulting in significant distortion of the estimation and a substantial increase in error. While the IA method is not directly affected by wheel speed failure, its inherent issue of integration error accumulation persists, causing the estimation curve to gradually deviate from the true value. In contrast, the MSF-FSM method automatically switches to the IMU-dominated mode when it detects that all wheel speeds are unreliable, effectively controlling error growth and demonstrating significantly better continuous estimation capability and dynamic response compared to the other methods. Table 3 further provides a specific numerical comparison of the maximum error, average error and RMSE for the four methods under this condition.

Based on the previous simulation results, the longitudinal speed estimation method proposed in this paper demonstrates excellent estimation performance under different slip conditions. Its maximum error and average error both outperform the other comparison methods. Even under the extreme condition of four-wheel full slip, this method can still maintain a high level of estimation accuracy, demonstrating strong environmental adaptability and robustness. The RMSE values further demonstrate the superiority of MSF-FSM. With an RMSE of only 0.35, it outperforms TA, WAT, and IA by a large margin, indicating that the proposed method not only has lower peak and mean errors but also exhibits much better estimation smoothness and consistency over time.

## 5. Hardware-in-the-Loop Testing and Analysis

### 5.1. HIL Testing Scheme

**Figure 13:**
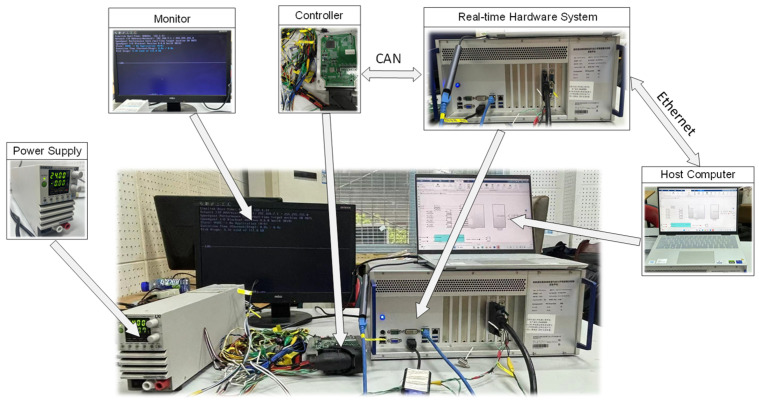
Real−Time Experimental Environment.

To verify the feasibility and deployment potential of the proposed longitudinal speed estimation method for distributed electric-drive loaders, a Hardware-in-the-Loop (HIL) experimental platform was built, as shown in Figure 13. The platform consists of core components including the host computer, real-time hardware system, and controller. The loader’s dynamic model is deployed on the Speedgoat real-time target machine, which is equipped with a 4.2 GHz quad-core processor and supports Ethernet, CAN, and high-speed I/O communication, enabling efficient execution of vehicle dynamics simulations.

The host computer exchanges data with the real-time hardware system via Ethernet, and the real-time system is connected to the controller through the CAN bus. After the control strategy is compiled on the host computer, it is downloaded to the controller for execution. The control signals output from the controller are received by the real-time hardware system through the CAN bus, thereby successfully establishing a closed-loop testing environment and providing a reliable experimental foundation for algorithm validation.

### 5.2. HIL Testing Analysis

**Figure 14:**
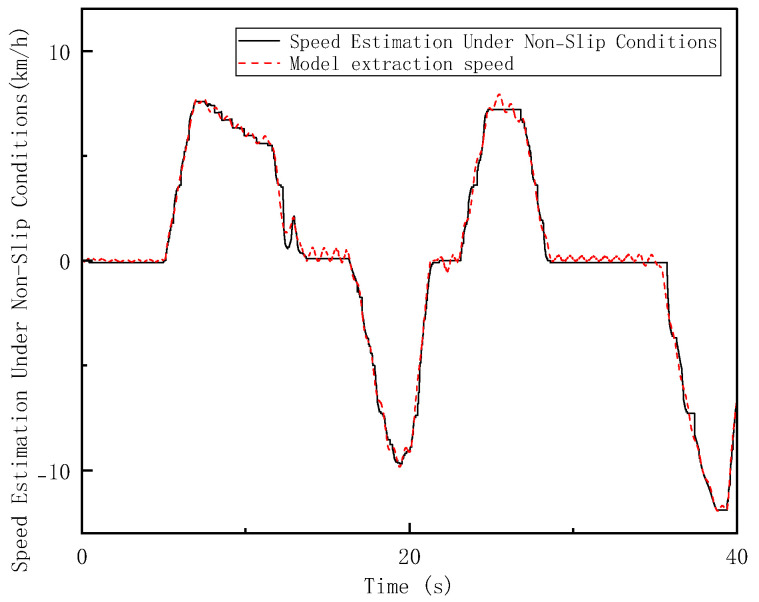
Vehicle Speed Estimation under Non−Slip Condition on High-Adhesion Surfaces in HIL Testing.

**Figure 15:**
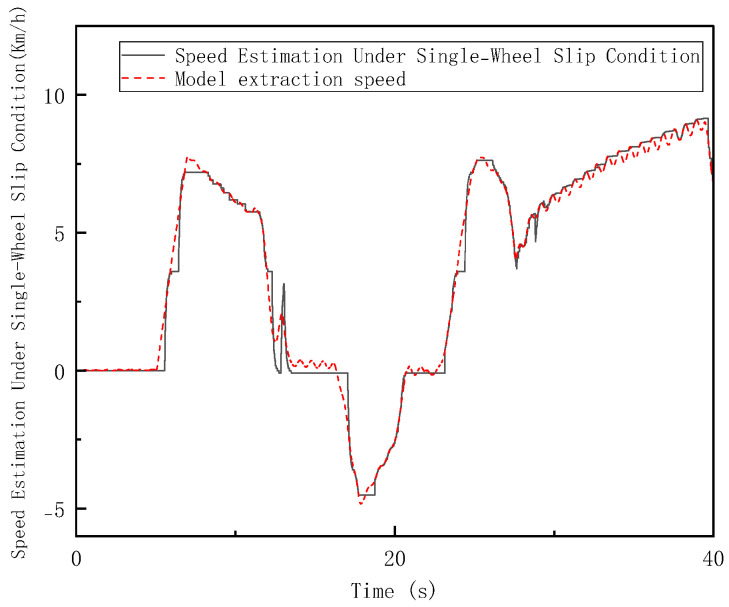
Vehicle Speed Estimation under Singl−Wheel Slip Condition on Low-Adhesion Surfaces in HIL Testing.

**Figure 16:**
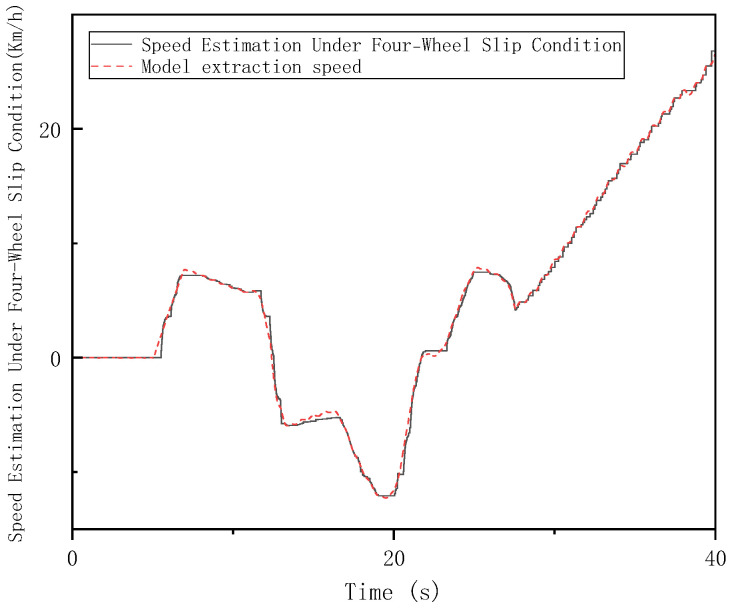
Vehicle Speed Estimation under Four−Wheel Slip Condition on Low−Adhesion Surfaces in HIL Testing.

**Figure 17:**
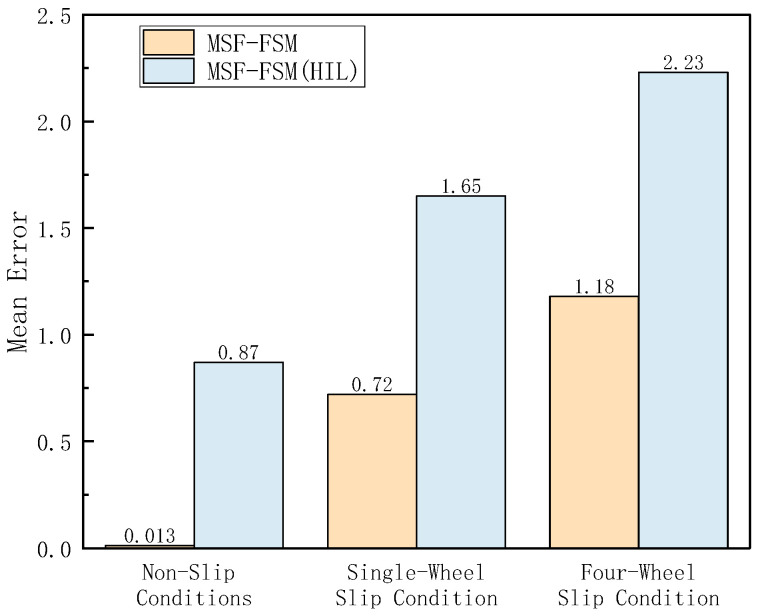
Maximum Vehicle Speed Estimation Error under Different Conditions in HIL Testing.

**Figure 18:**
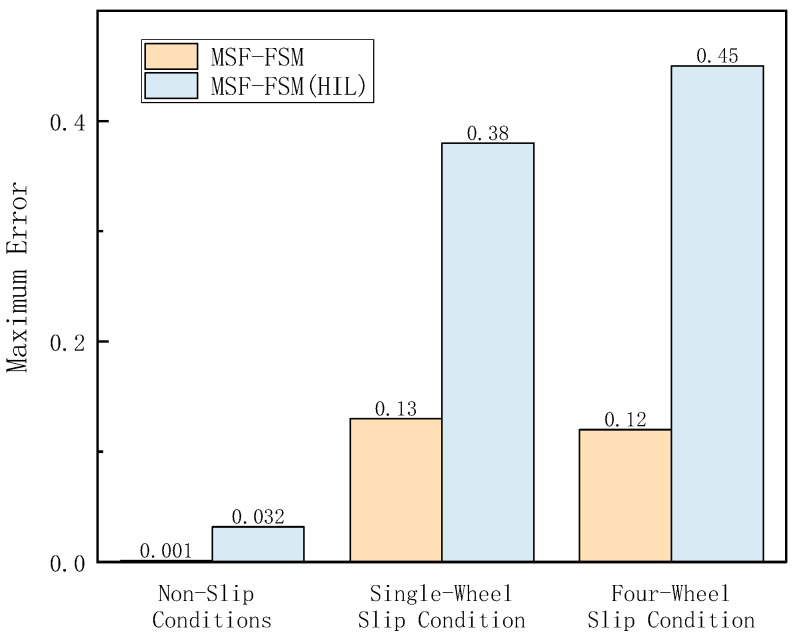
Average Vehicle Speed Estimation Error under Different Conditions in HIL Testing.

In the Hardware-in-the-Loop (HIL) testing, high-adhesion road conditions consistent with the simulation scenario were set. The loader was tested under the V-type operation cycle on a high-adhesion surface with an adhesion coefficient of 0.9 (non-slip condition), a low-adhesion surface with an adhesion coefficient of 0.35 (single-wheel slip condition), and a four-wheel slip condition. As shown in Figure 14, Figure 15 and Figure 16, the results of the longitudinal speed estimation in both the HIL and simulation tests are consistent, with minimal error. The slight differences can be attributed to sensor signal acquisition errors and mechanical constraints in the hardware-in-the-loop system. These results effectively validate the feasibility and reliability of the proposed FSM-based multi-sensor fusion speed estimation strategy for practical deployment. The maximum and average errors for the three test conditions are shown in Figure 17 and Figure 18.

## 6. Conclusions

This paper proposes a longitudinal speed estimation method with good real-time performance for distributed electric-drive loaders. The method is based on a Finite State Machine (FSM) and designs an adaptive speed estimation strategy. This strategy uses a dual-threshold judgment based on wheel acceleration and linear velocity to identify tire slip conditions. For the identified “non-complete slip” and “complete slip” states, the method applies the weighted average of reliable wheel speeds and the IMU acceleration integration method for speed estimation. The former fully utilizes effective wheel speed information through credibility weighting, while the latter effectively overcomes the integration drift problem through short-term integration and initial value updating. Additionally, the articulated steering projection method compensates for the geometric errors caused by articulated steering, enabling accurate and reliable longitudinal vehicle speed estimation under complex working conditions. Simulation results show that, compared to simple averaging (TA), selective averaging (WAT), and pure integration (IA) methods, the proposed method (MSF-FSM) achieves over 75% improvement in speed estimation accuracy under low-adhesion road conditions such as snow and ice, fully demonstrating its superiority.

Based on this, the engineering feasibility of the strategy was further validated through Hardware-in-the-Loop (HIL) testing. The loader’s dynamic model and the proposed speed estimation strategy were successfully deployed, and the results show that the FSM-based multi-sensor fusion method operates stably and reliably in practical systems, with good deployment potential.

Despite the achievements presented in this study, several limitations remain, and future research directions are proposed accordingly. First, the thresholds used for identifying wheel slip conditions are currently fixed calibration values. Future work may introduce an adaptive threshold mechanism that dynamically adjusts the decision criteria based on real-time road adhesion coefficients, thereby further improving the robustness and estimation accuracy under complex operating conditions. Second, when the vehicle undergoes prolonged continuous slipping on low-adhesion roads, the IMU acceleration integration method still accumulates drift errors over time, which cannot be completely eliminated. Therefore, the proposed method is more suitable for scenarios involving short-term extreme slip or intermittent adhesion recovery. For cases of extremely long-duration continuous slip, future studies may consider incorporating external reference information for auxiliary correction. Furthermore, the validation scenarios in this paper are mainly focused on uniform adhesion coefficient road surfaces. For complex working conditions commonly encountered in construction machinery, such as step changes in the adhesion coefficient (μ-jump) and different adhesion levels on the left and right sides (split-μ surfaces), the robustness of the proposed algorithm has not yet been verified. Future work will conduct further research based on hardware-in-the-loop simulation and consider optimizing the state machine strategy to improve transient response performance. Finally, this study simplifies the effect of vibration on the IMU by assuming that vibration noise can be largely suppressed by conventional low-pass filtering and that short-term integration can mitigate high-frequency disturbances to some extent. The complex longitudinal and lateral vibrations under loader shoveling and slip conditions have not been fully considered regarding their impact on estimation accuracy. Future work will investigate vibration mitigation measures such as adaptive filtering or multi-IMU redundancy compensation using real-world vibration data to further improve robustness.

## Figures and Tables

**Table 1:** State Transition Table.

Current State	Event	Next State
Non-completely slipping state	At least one trustworthy wheel is detected within the time window	Non-completely slipping state
Non-completely slipping state	No trustworthy wheel is detected at any moment within the time window, and a sharp change in wheel speed or IMU signals is detected (which may indicate the onset of slipping), causing the window to be temporarily shortened for a quick response	Completely slipping state
Completely slipping state	A trustworthy wheel is detected within the window, and if the window was previously shortened, it is restored to the original length	Non-completely slipping state
Completely slipping state	No trustworthy wheel is detected at any moment within the time window	Completely slipping state

**Table 2:** Vehicle Speed Estimation Error under Single-Wheel Slip Condition.

Method	Maximum Error	Average Error	RMSE
TA	221	28.77	38.78
WAT	7.55	0.33	0.97
IA	1.38	0.52	0.7
MSF-FSM	0.72	0.13	0.17

**Table 3:** Vehicle Speed Estimation Error under Four-Wheel Slip Condition.

Method	Maximum Error	Average Error	RMSE
TA	235.02	37.86	57.48
WAT	226.93	27.61	42.26
IA	2.4	0.923	1.21
MSF-FSM	1.18	0.12	0.35

## Data Availability

Data are available from authors on reasonable request.

## Abbreviations

The following abbreviations are used in this manuscript:

FSM	Finite State Machine
TA	Simple average method based on the four-wheel wheel speed
WAT	Selective averaging method based on threshold that weights the trustworthy wheel speeds after detecting the slipping state
IA	Estimation method based on IMU acceleration integration
MSF−FSM	The FSM-based multi-sensor fusion method
HIL	Hardware-in-the-Loop
